# [^68^Ga]Ga-FAP-2286—Synthesis, Quality Control and Comparison with [^18^F]FDG PET/CT in a Patient with Suspected Cholangiocellular Carcinoma

**DOI:** 10.3390/ph17091141

**Published:** 2024-08-29

**Authors:** Anton Amadeus Hörmann, Gregor Schweighofer-Zwink, Gundula Rendl, Kristina Türk, Samuel Nadeje, Kristina Haas, Theresa Jung, Ursula Huber-Schönauer, Lukas Hehenwarter, Mohsen Beheshti, Christian Pirich

**Affiliations:** Department of Nuclear Medicine and Endocrinology, University Hospital Salzburg, Paracelsus Medical University, 5020 Salzburg, Austrial.hehenwarter@salk.at (L.H.);

**Keywords:** gallium-68, FAP, automated synthesis, PET/CT, FDG-negative cancer, imaging cholangiocellular carcinoma

## Abstract

[^68^Ga]Ga-FAP-2286 is a new peptide-based radiopharmaceutical for positron-emission tomography (PET) that targets fibroblast activation protein (FAP). This article describes in detail the automated synthesis of [^68^Ga]Ga-FAP-2286 using a commercially available synthesis tool that includes quality control for routine clinical applications. The synthesis was performed using a Scintomics GRP-3V module and a GMP grade ^68^Ge/^68^Ga generator. A minor alteration for transferring the eluate to the module was established, eliminating the need for new method programming. Five batches of [^68^Ga]Ga-FAP-2286 were tested to validate the synthesis. A stability analysis was conducted up to 3 h after production to determine the shelf-life of the finished product. The automated synthesis on the Scintomics GRP-3V synthesis module was found to be compliant with all quality control requirements. The shelf-life of the product was set to 2 h post-production based on the stability study. A patient suffering from cholangiocellular carcinoma that could not be clearly detected by conventional imaging, including a [^18^F]FDG-PET/CT, highlights the potential use of [^68^Ga]Ga-FAP-PET/CT.

## 1. Introduction

To date, [^18^F]Fluorodeoxyglucose ([^18^F]FDG) is increasingly a ‘gold standard’ for cancer imaging using positron-emission tomography (PET), not only due to its high impact in patient management but also due to its widespread availability and a reduction in its cost in recent years. However, [^18^F]FDG itself has its limitations for certain cancer entities, e.g., in mucinous neoplasms and hepatocellular carcinomas [1].

A very promising novel group of PET imaging agents are small molecules targeting the fibroblast activation protein (FAP). FAP is a type II integral membrane serine protease, which is hardly expressed in healthy tissues [2]. However, FAP is significantly and detectably expressed by cancer-associated fibroblasts (CAFs), promoting tumorigenesis and malignant behavior. CAFs interact with cancer cells via multiple mechanisms and support tumors by enhancing growth and evading immune response [3,4]. A multitude of signals are generated that actively encourage cell proliferation, the development of metastases, and even the establishment of treatment resistance [5].

**Figure 1:**
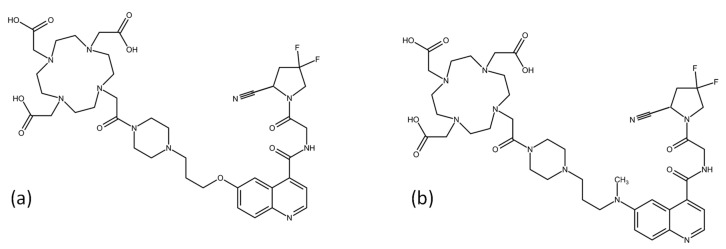
Chemical structure of (**a**) FAPI-04 and (**b**) FAPI-46.

FAP expression was found on the surface of fibroblasts in the stroma of 90% of different cancers including breast, colorectal, bladder, lung and ovarian [6]. The initial development of FAP inhibitors (FAPI) based on the *N*-(4-quinolinoyl)glycyl-(2-cyanopyrrolidine) moiety led to further-optimized, quinoline-based FAP inhibitors, most notably 2,2′,2″,2‴-(1,4,7,10-tetraazacyclododecane-1,4,7,10-tetrayl)tetraacetic acid (DOTA)-conjugated FAPI-04 and FAPI-46, allowing radiolabeling with different radionuclides for theranostic applications [7,8,9]. The chemical structures are shown in Figure 1.

**Figure 2:**
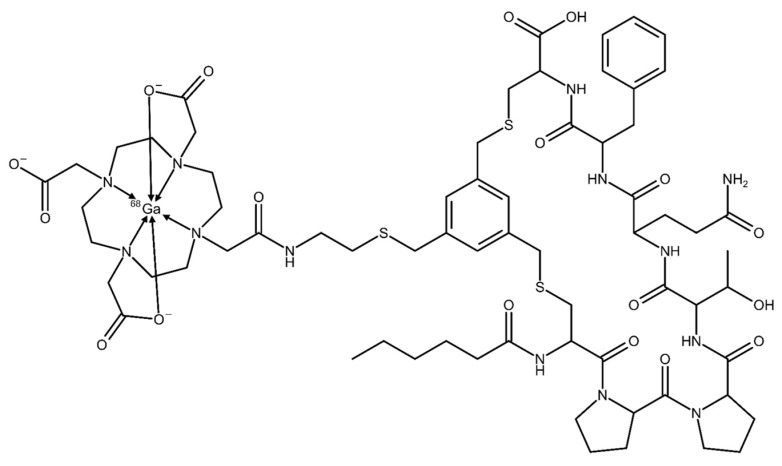
Chemical structure of [^68^Ga]Ga-FAP-2286.

For therapeutic use, however, tumor retention of the radiopharmaceutical is of great importance in order to deliver a maximum dose to the tumor site. To overcome the problem of FAPI-04 showing a very short retention time, the peptide-based cyclic FAP-binding molecule FAP-2286 was developed, combining high affinity and enhanced retention time in FAP-positive tumors in mice and in first-in-human results when radiolabeled with lutetium-177 [10,11]. The chelator DOTA also allows gallium-68 radiolabeling, making FAP-2286 an excellent candidate peptide-based theranostic agent. The increased interest in novel radiotracers is encouraging the implementation of simple and reliable automated synthesis methods of gallium-68 radiopharmaceuticals to provide diagnostic capabilities in circumstances where the [^18^F]FDG scan has been negative or inconclusive and consequently to provide an improved range of therapeutic options. In Figure 2, the chemical structure of [^68^Ga]Ga-FAP-2286 is displayed. 

The automated synthesis and quality control of [^68^Ga]Ga-FAP-2286 has not yet been described in detail. In this article, the process of the synthesis and the quality control of [^68^Ga]Ga-FAP-2286 using a commercially available synthesis module in combination with a ^68^Ge/^68^Ga generator according to current good radiopharmaceutical practice guidelines of the EANM is described [12]. 

To further highlight the significance of using this novel class of radiopharmaceuticals, a clinical case from our department with a high suspicion of a liver tumor but inconclusive conventional imaging, including a negative [^18^F]FDG-scan, is provided.

## 2. Results

### 2.1. Radiolabeling and Quality Control of [^68^Ga]Ga-FAP-2286

The automated synthesis of [^68^Ga]Ga-FAP-2286 used the configuration and set-up of the synthesis module described in the method section and was performed within a total synthesis time of approximately 35 min. On average, the generators yielded ~65% of the theoretically available gallium-68 activity.

The reference standard [^non-radioactive^Ga]Ga-FAP-2286 was successfully labeled, confirmed by a shift in HPLC retention time (R_t_ = 6.14 min) versus FAP-2286 alone (R_t_ = 4.80, see Figure S1), which was additionally confirmed by ESI-MS analysis (MW calculated (*m*/*z*) [M+H]^+^: 1537.48; MW found (*m*/*z*) [M+H]^+^: 1536.21). In Figures S2 and S3, the UV–Vis chromatogram of [^non-radioactive^Ga]Ga-FAP-2286 and the ESI-MS spectrum of [^non-radioactive^Ga]Ga-FAP-2286 are shown, respectively.

Five batches of [^68^Ga]Ga-FAP-2286 were produced, with a total activity of 345.2 ± 80.2 MBq of final product, corresponding to an average decay-corrected yield of 71.8 ± 1.9% based on the initial gallium-68 activity measured before starting the synthesis. The percentage of the peak corresponding to [^68^Ga]Ga-FAP-2286 determined by radio-HPLC was 96.2 ± 1.2%. Only slightly higher amounts of hydrophilic impurities with lower retention times could be detected. The addition of H_2_O_2_ (3% solution) to the quality control sample showed an increase in hydrophilic impurities after re-injection to the HPLC system (see Figure S4). The identity of [^68^Ga]Ga-FAP-2286 was determined by comparing the retention time in the UV–Vis of the cold standard [^non-radioactive^Ga]Ga-FAP-2286 with the retention time of the radiodetector signal of [^68^Ga]Ga-FAP-2286 (R_t_ = 6.39 min). Both peaks showed similar retention times and were well within the limit of relative retention times (RRT) of between 0.9 and 1.1, confirming the identity of [^68^Ga]Ga-FAP-2286.

**Figure 3:**
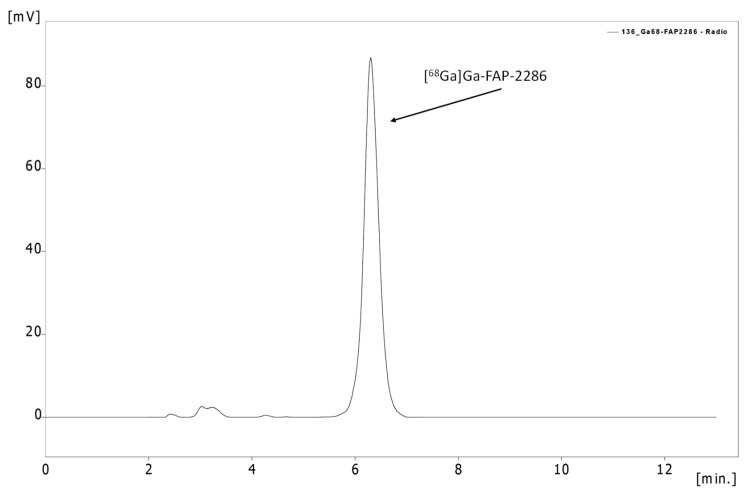
Exemplary radio-HPLC chromatogram of [^68^Ga]Ga-FAP-2286.

In Figure 3, an exemplary radio-HPLC chromatogram of [^68^Ga]Ga-FAP-2286 post-production (p.p.) is shown. The corresponding UV-Chromatogram is shown in the Supporting Information (Figure S6).

**Figure 4:**
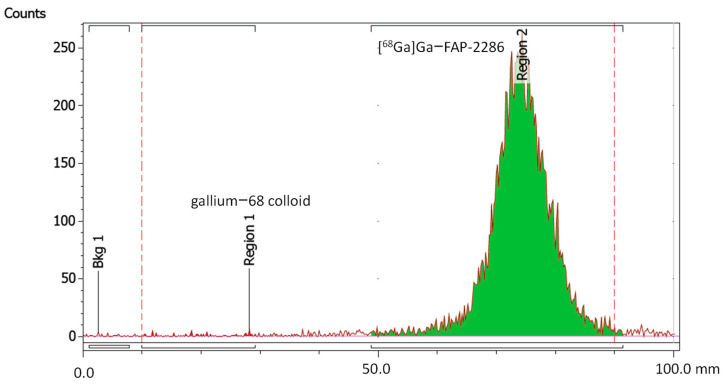
iTLC chromatogram of [^68^Ga]Ga-FAP-2286.

Radionuclide incorporation with R_f_ value of 0.5–1.0 based on radio-iTLC measurements using a 1:1 (*v*/*v*) mixture of 1 M ammonium acetate and methanol as mobile phase was 99.9 ± 0.1%. No colloidal gallium-68 species was found with R_f_ value of 0.0–0.3 with values below the limit of quantification (LOQ = 0.3 kBq/µL). In Figure 4, an exemplary iTLC chromatogram of [^68^Ga]Ga-FAP-2286 is shown.

For all synthesized batches, the test method for HEPES content described in Ph. Eur. for other gallium-labeled radiopharmaceuticals showed clearly lower intensity of the spot, with an R_f_ value of ~0.2 after staining with I_2_ gas. Consequently, the HEPES content of the final product is less than the 500 µg/V limit stated in the Ph. Eur.

**Figure 5:**
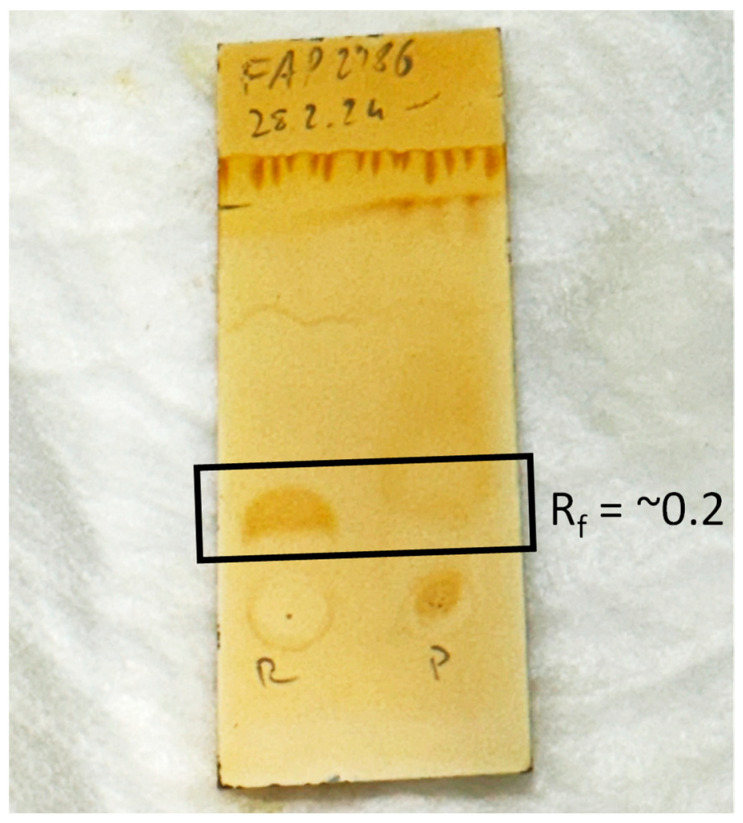
Picture of an iodine-stained iTLC for determination of the HEPES content in the final product (R = HEPES reference solution; P = final product).

Figure 5 shows a picture of an exemplary iTLC plate for the determination of the HEPES content after staining with I_2_ vapor for 4 min. Table 1 summarizes the results obtained for all batches, and in Table S1, the overall radiochemical purity and radiochemical yield from the single batches are shown.

### 2.2. Stability Analysis of [^68^Ga]Ga-FAP-2286

The stability of the final product was tested up to 3 h p.p. by radio-HPLC and iTLC analysis in additional batches (*n* = 3). The RCP of the preparation was 98.8 ± 0.1%, and the formation of colloidal did not exceed 1% after 3 h p.p.

The overall radiochemical purity was 98.1 ± 0.0% after 3 h p.p. The stability data are shown in Table 2.

The formation of hydrophilic impurities was not prevented by the effect of changes during the radiolabeling process. The addition of ethanol to the radiolabeled solution resulted in further impurity formation and just met the acceptance criteria (see Figure S5). In Table 3, the results from the radio-HPLC, iTLC and pH measurements are shown.

### 2.3. [^68^Ga]Ga-FAP-2286-PET/CT Scans and Further Clinical Outcome

Initial PET/CT scan with [^68^Ga]Ga-FAP-2286 showed an intensively focal uptake in the porta hepatis in projection to the bile duct drainages, which has been assessed as a malignant tumor and the primary cause of the patient’s cholestasis. We also concluded that there is no evidence of metastasis.

**Figure 6:**
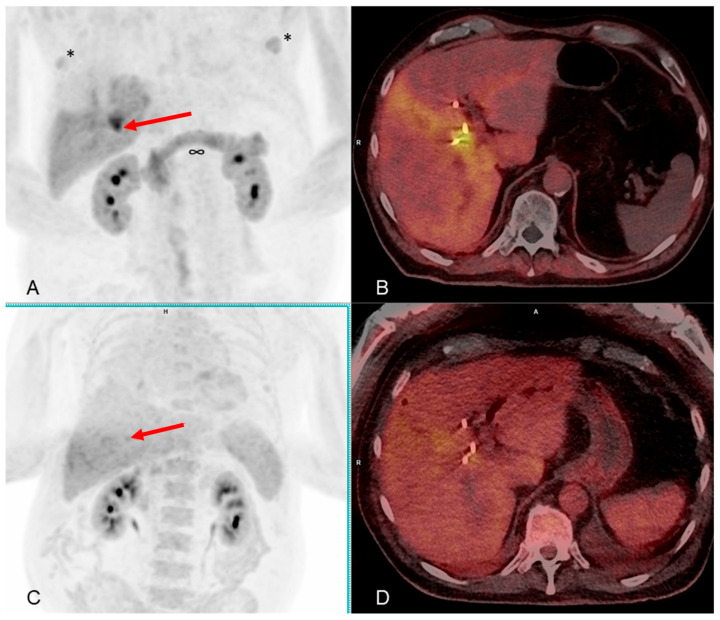
(**A**,**B**) MIP and hybrid PET/CT scan slice from December 2023 using [^68^Ga]Ga-FAP-2286: the red arrow marks an intense focal tracer uptake, which has been interpreted as the primary tumor lesion atop the confluence of the right and left hepatic bile ducts (Klatskin tumor). SUV_max_ 13.7; SUV_peak_ 10.7. (**C**,**D**) MIP and hybrid PET/CT scan slice from November 2023 using [^18^F]FDG: the red arrow marks a slight, inhomogeneous background noise enhancement at the confluence of the right and left hepatic bile ducts. However, the entire liver tissue shows this inhomogeneous uptake pattern. There is no clear focally-increased, neoplastic uptake delineated. Please take note of the liver parenchyma’s unevenly dispersed FAP expression, which has been interpreted as reactive uptake after acute cholangitis. It is important to take attention of the diffuse expression of FAP in the pancreas (∞), which has been attributed to a reactive inflammatory process following ERCP pancreatitis. Uptake in gynecomastic tissue is indicated by the *.

Figure 6 shows the negative [^18^F]FDG scan compared to the intensively focal uptake seen at the initial [^68^Ga]Ga-FAP-2286-PET/CT scan in December 2023.

Additional findings of diffuse uptake in the pancreas and in parts of the right liver lobe have been interpreted as reactive findings due to inflammation after the bile duct ste-nosis and several ERCPs. Notably, there is degenerative uptake to the hips and demarcation of a gynecomastia on both sides as non-cancer-specific sites of tracer uptake in benign disorders.

**Figure 7:**
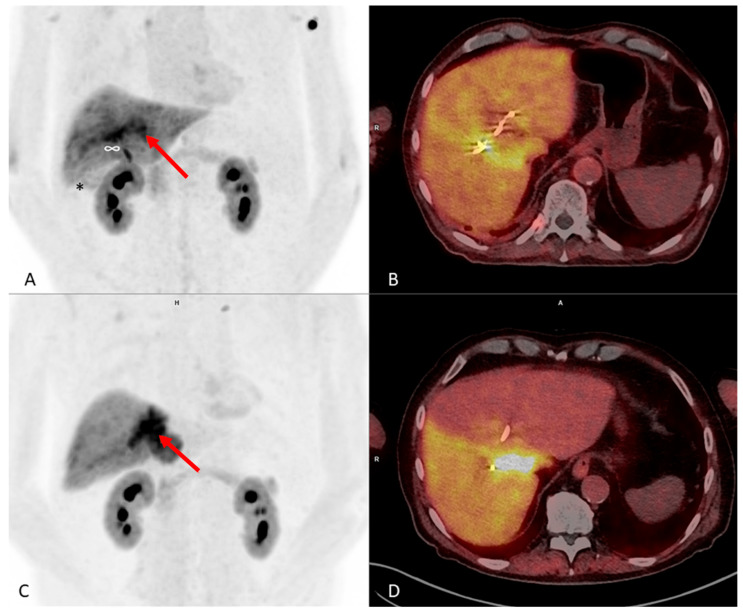
(**A**,**B**) MIP and hybrid PET/CT scan slice from June 2024 using [^68^Ga]Ga-FAP-2286: the red arrow marks the primary tumor lesion with a decreasing size and tracer uptake as compared to (**C**,**D**) after change of chemotherapy to FOLFOX. SUV_max_ 15.6; SUV_peak_ 13.6. (**C**,**D**) MIP and hybrid PET/CT scan slice from March 2024 using [^68^Ga]Ga-FAP-2286: the red arrow marks the primary tumor lesion with an increasing size and tracer uptake at this time point after chemotherapy with cisplatin, gemcitabine, and durvalumab. SUV_max_ 24.6; SUV_peak_ 20.5. Please note a new uptake in image A, which has been interpreted as diffusely reactive after acute cholangitis with cholezystitis (*) that has been treated with antibiotics at the time point of the PET scan. Additionally, there is a new uptake at the proximal common bile duct (∞), as well as diffuse linear-shaped uptake along the central bile ducts and a change of diffuse FAP expression in the liver parenchyma, considered as reaction to the inflammatory process.

A chemo-immunotherapy was started using cisplatin, gemcitabine, and durvalumab. During follow-up using [^68^Ga]Ga-FAP-2286-PET/CT and tumor markers, disease progression occurred, leading to a change of treatment to a FOLFOX scheme as a second-line therapy. Consecutively, a significant drop of tumor markers could be observed, and the most recent restaging [^68^Ga]Ga-FAP-2286-PET/CT scan showed a partial remission. Figure 7 shows the imaging results of the two recent [^68^Ga]Ga-FAP-2286-FAP-PET/CT scans. The patient had a partial remission of the primary tumor. Yet, at the time point of the most recent restaging, he presented again with acute cholangitis and cholezystitis, needing antibiotic treatment and the renewal of the bile duct drainages via ERCP.

## 3. Discussion

Using radiopharmaceuticals to target CAFs via gallium-68-labeled small compounds, such FAPI-04 or FAPI-46, is a unique and promising approach to cancer diagnostics, which showed superior uptake in different cancer entities compared to [^18^F]FDG [13,14,15,16]. The majority of this new class of radiopharmaceuticals are conjugated to the bifunctional chelator DOTA, which enables radiolabeling not only with imaging radionuclides such as gallium-68 but also with therapeutic radionuclides such lutetium-177. The short-lasting retention period of FAPI-04 or FAP-46 in the microenvironment does not affect imaging quality but is a major drawback for therapeutic purposes. However, the novel cyclic peptide-based FAP analogue, FAP-2286, showed a significantly slower clearance from the stroma of the tumor tissue, as seen in xenografted SCID beige mice, when radiolabeled with lutetium-177 compared to [^177^Lu]Lu-FAPI-46 [11]. Therefore, the use of FAP-2286 in the clinical setting is of great interest and can not only be used for the imaging of malignant tumors but also to take advantage of the ‘matched pair’ of gallium-68 and lutetium-177 for possible future therapeutic interventions. Furthermore, encouraging results from preclinical and clinical studies justify further clinical evaluation of [^68^Ga]Ga-FAP-2286 in cancer diagnostics and the availability of a simple synthesis procedure [10,11,17].

In this study, we investigated the automated synthesis on a commercially available synthesis module of [^68^Ga]Ga-FAP-2286 for PET/CT imaging in patients with solid tumors.

The automated synthesis on a Scintomics module and the quality control of [^68^Ga]Ga-FAP-2286 were not described in detail until now, having only been mentioned by Banihashemian et al. [18]. Phlak et al. recently published the automation of the synthesis of [^68^Ga]Ga-FAPI-46 on the same commercially available synthesis module using a simultaneous elution of two ^68^Ge/^68^Ga generators by a self-programmed synthesis method [19]. However, this process requires a dedicated software tool and knowledge in programming thereof. The method described in detail in this article allows the use of the synthesis method provided by the company and the performance of a simple and rapid synthesis with a minimum of manual intervention.

The addition of 5 mL 0.1 M HCl to the eluate had no influence on the decay corrected radiochemical yield due to the strong cation exchange cartridge which eliminates hydrochloric acid present in the eluate and concentrates [^68^Ga]Ga^3+^ ions (71.8 ± 2.1% vs. 72.6 ± 4.9% as reported by Phlak et al.) and which was also previously described by Da Pieve et al. [19,20].

The use of HEPES, one of the ‘Good´s buffers’, is one of the buffers of choice for gallium-68 labeling due to its weak metal complexing character, preventing the formation of gallium-68 colloidal species [21,22,23]. However, the Ph. Eur. (Edition 11.0) lists HEPES as an impurity with a maximum injected amount of 500 µg/V and does not authorize it for human injection. Intravenous injection of HEPES in dogs found no adverse effects up to 520 mg/kg body weight/day, and in rats showed no mortality up to doses of 2000 mg/kg body weight [24,25]. The amount used during radiolabeling is much lower. Furthermore, an additional purification step during automated synthesis addresses this problem to ensure that the radiopharmaceutical is safe for human application after the automated synthesis process. Nevertheless, quantification is required prior to intravenous injection. Alternative methods to the Ph. Eur. method have been developed to quantify the HEPES content in the final product, including HPLC analysis and iTLC measurements, due to unreliable results [26,27,28]. This requires an additional HPLC system dedicated to HEPES testing or testing after radiochemical purity analysis, which is not feasible due to gallium-68’s short half-life. In this study, the method provided by the Ph. Eur. gave clear and reliable results without the need of optical determination measurements, which eliminates the need to change to other methods or take additional validation steps.

Due to the long synthesis time of approximately 35 min, the need for fast quality control becomes much more important, especially when using an older ^68^Ge/^68^Ga generator providing lower activities. The development of a fast radio-HPLC analysis with a total run time of 13 min gave a good compromise between analytical performance and run time. The limiting factor, however, is the determination of the HEPES content. This requires preparation before the synthesis, e.g., the application of the reference spots. Overall, the duration of the quality control in a well-organized team can be set to a minimum total analysis time of approximately 13 min, as the radio-HPLC run requires the longest analysis time of 13 min.

Radio-HPLC analysis of the final product showed some minor hydrophilic impurities, which could not decreased using a labelling temperature 95 °C instead of 125 °C.

However, the formation of the hydrophilic impurities be increased with the oxidizing agent H_2_O_2_, which may be occur due to the oxidation of the thioether groups within the structure to sulfoxides or even sulfones. The addition of antioxidants or radical scavengers, such as ascorbic acid (AA) in different amounts (10 and 50 mg), or the addition of ethanol (20% *v*/*v* total concentration) to the labelling solution did not prevent the formation of these impurities either, as has been seen with other radiopharmaceutical preparations and in particular with lutetium-177 radiopharmaceuticals [29,30,31,32]. As all quality parameters were met during the validation process, no further reagents were added to the labelling mixture or the final product, nor were any changes made to the provided validated synthesis method.

## 4. Materials and Methods

### 4.1. Materials

FAP-2286 for validation of the automated synthesis and quality control was purchased from MedChemExpress (1 Deer Park Dr, Suite Q, Monmouth Junction, NJ, USA). For patient use, FAP-2286 in GMP quality was obtained from piCHEM (Raaba-Grambach, Austria) in lyophilized aliquots of 50 µg net peptide. A Scintomics GRP-3V module (Scintomics, Fürstenfeldbruck, Germany) was used for the automated synthesis of [^68^Ga]Ga-FAP-2286 in combination with a commercially available labeling kit consisting of a sterile disposable cassette for gallium-68 peptides (SC-01-H) and a set of reagents (SC-01) (ABX, Radeberg, Germany). Sterile filtered 96% ethanol solution, ascorbic acid and Pascorbin^®^ (7.5 mg ascorbic acid in 50 mL water for injection) were contributed by the hospital pharmacy.

Mobile phases for high-performance liquid chromatography (HPLC) were purchased as HPLC-grade solvents (Carl Roth GmbH & Co. KG, Karlsruhe, Germany). The [^68^Ga]GaCl_3_ solution was obtained from a ^68^Ge/^68^Ga generator in GMP quality with a total activity of germanium-68 of 1850 MBq at the time of calibration by eluting with 0.1 M HCl solution built within the generator (Galli Ad, IRE EliT, Fleurus, Belgium). Sterile 0.1 M hydrochloric acid as an additional reagent in the synthesis and a 1:1 mixture of 1 M ammoniumacetate:methanol for instant thin-layer chromatography (iTLC) was provided by the hospital pharmacy. 

The reference standard [^non-radioactive^Ga]Ga-FAP-2286 was prepared by pipetting 50 µL (50 µg) FAP-2286, 20 µL GaBr_3_ (Merck, Darmstadt, Germany; 4.4 molar excess, 2.3 mg/mL in 0.1 M HCl), and 10 µL of a 1.14 M sodium acetate trihydrate solution (Merck, Darmstadt, Germany; 310 mg in 2 mL H_2_O) in a low protein Eppendorf^®^ tube (Eppendorf SE, Hamburg, Germany). The reaction mixture was heated in a boiling-water bath for 10 min. To remove any excess of free gallium after labeling, a SepPak^®^ tC18 Plus Light (Waters Corporation, Milford, MA, USA), after conditioning with 5 mL ethanol and 5 mL H_2_O, was used by washing the cartridge with 10 mL H_2_O. The labeled peptide was then eluted using 1 mL 50% ethanol solution. Successful determination of the labeling process was confirmed by electrospray ionization mass spectrometry (ESI-MS) experiment using a LCMS-2050 Nexera (Shimadzu, Korneuburg, Austria) with the following conditions: Scan range (*m*/*z*) 200.0–2000.0; sampling 500 ms/2 Hz; mobile phase 30:70 water:acetonitirile (*v*/*v*) + 0.1% formic acid; flow rate 0.5 mL/min. Data were acquired and evaluated with LabSolutions software (Shimadzu, Korneuburg, Austria; version 5.120) and a shift in HPLC retention time of the UV–Vis signal. For injection to the HPLC system, the solution was diluted 1:10 with water.

### 4.2. Radiolabeling of [^68^Ga]Ga-FAP-2286

Radiolabeling was performed in a Class A laminar airflow safety cabinet with lead glass shielding in a Class D cleanroom background environment. Strict microbial monitoring of the environment and personnel was carried out during the synthesis. 

Prior to starting the automated synthesis and elution of the ^68^Ge/^68^Ga generator, FAP-2286 was dissolved in 1.3 mL of 1.5 M 4-(2-hydroxyethyl)-1-piperazineethanesulfonic acid (HEPES) buffer and then transferred to the reactor vial. The reactor vial was then placed in the heating block and connected to the line from valve 2 (red screw, vertical) and the line from valve 12 (blue screw, vertical). 

**Figure 8:**
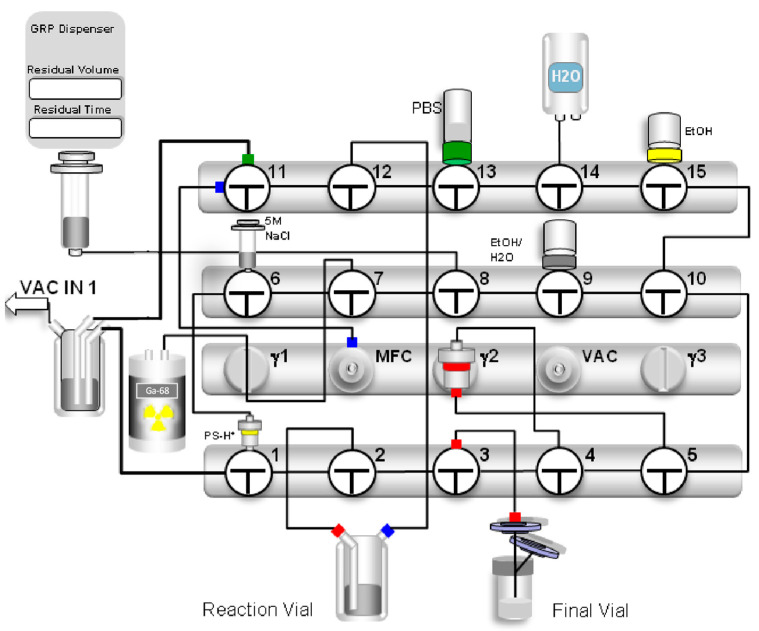
Scheme of the automated cassette-based production of [^68^Ga]Ga-FAP-2286 using the GRP-3V synthesis module.

A 10 mL vial of 96% ethanol (5 mL, valve 15 vertical), a 10 mL vial of 50% ethanol (2 mL, valve 9 vertical), and a 20 mL vial of phosphate-buffered saline (PBS, valve 13 vertical) were attached to the cassette. The product vial was assembled with a 0.2 µm Millex^®^ hydrophobic vent filter unit (Merck, Darmstadt, Germany) and a 0.22 µm Cathivex^®^-GV low-protein binding sterile filter (SLGV02505, Merck Millipore, Cork, Ireland) and connected to the product line (valve 3 vertical). A polyethylene extension catheter (Vygon GmbH & Co. KG, Aachen, Germany) was used to connect the module to the eluate vial (valve 7 vertical). Two waste lines were connected to valves 1 and 11 (both horizontal). The PS-H^+^ cartridge was mounted on valve 1 (vertical) and connected to the line from valve 6 (horizontal). For the N_2_ gas supply, the line from valve 11 (horizontal) was connected to the mass flow control (MFC) port. The water for the injection bag was connected to the line from valve 14 (vertical). By way of illustration, the schematic of the automated synthesis module is shown in Figure 8.

The synthesis starts with the manual elution of the generator by turning the rotary knob 90° on the generator for 20–30 s. The knob was then returned to the starting position, and the [^68^Ga]GaCl_3_ eluate was eluted by connecting the generator line to an external vacuum vial for 3 min. At the end of the transfer process, the generator line was removed from the vacuum vial, and the vial was measured in the dose calibrator for calculation of the radiochemical yield. The vial was placed back in the lead shielding, and a Millex^®^ hydrophobic vent filter was connected. To ensure sufficient volume for the transfer to the module using the built-in syringe, 5 mL of sterile 0.1 M HCl was added to the elution vial. After connecting the vial to the module, the automated synthesis was started by clicking the ‘Start’ button on the module software. The first step of the fully automated process was to pre-condition the SepPak^®^ Light C18 cartridge (Waters, Milford, MA, USA) with ethanol and water, followed by a drying step with N_2_ gas. The 20 mL syringe on the module was used to transfer the [^68^Ga]GaCl_3_ eluate from the vial to the Chromafix^®^ PS-H^+^ solid phase extraction (SPE) cartridge (Marchery-Nagel, Düren, Germany). Excess hydrochloric acid was discharged to the waste. [^68^Ga]GaCl_3_ was then eluted from the PS-H^+^ SPE cartridge and transferred into the reaction vial containing the buffer and FAP-2286 using 1.5 mL of 5 M NaCl. Radiolabeling was performed at 125 °C for 6 min. To remove any free gallium-68 or colloidal gallium-68 species after radiolabeling, the reaction mixture was loaded onto a SepPak^®^ C18 cartridge. The reactor was then washed two times with water and loaded on the cartridge. After transferring the reaction solution onto the cartridge and subsequently performing two washing steps with water, the product was eluted with 2 mL of 50% ethanol through a sterile filter into the final product vial and diluted with 15 mL PBS.

### 4.3. Quality Control of [^68^Ga]Ga-FAP-2286

The European Pharmacopoeia monographs ‘Gallium (^68^Ga) Edotreotide Injection’ (2482), ‘Gallium (^68^Ga) PSMA-11 Injection’ (3044), ‘Gallium (^68^Ga) oxodotreotide injection (3050)’, and ‘Gallium (^68^Ga) DOTANOC injection (3051)’ were used for the quality criteria of the final solution of [^68^Ga]Ga-FAP-2286, since those are the only monographs for gallium-labeled radiopharmaceuticals currently available (Ph. Eur. version 11.3).

HPLC analysis of [^68^Ga]Ga-FAP-2286 generated from the automated synthesis was performed using a Scintomics chromatography consisting of an binary pump (SCI 8111) and degasser (SCI 8101) and equipped with a variable UV-detector SCI 8120 (UV–Vis = 254 nm) and a HERM LB 500 NaI-radiodetector (Berthold Technologies GmbH & Co., Bad Wildbad, Germany). An Onyx Monolithtic C18 100 mm × 4.6 mm (Phenomenex, Aschaffenburg, Germany) was used together with a flowrate of 2 mL/min and the following gradient: water/0.1% trifluoroacetic acid (TFA) (A) and acetonitrile/0.1% TFA (B): 0–2 min, 25% B; 2–8 min, 25–28% B; 8–8.01 min, 28–80% B; 8.01–10 min, 80% B; 10–10.1 min, 80–25% B; 10.01–13 min, 25% B. Clarity (Version 6.2.0.208) was used for evaluation of the HPLC chromatograms.

The identity of [^68^Ga]Ga-FAP-2286 was determined by comparing the retention time of the radio-HPLC signal of the radiolabeled product with the UV–Vis retention time of unlabeled FAP-2286 and FAP-2286 labeled with the stable isotope of gallium ([^non-radioactive^Ga]Ga-FAP-2286).

The radiochemical purity (RCP) was quantified by integrating the area of the main peak corresponding to [^68^Ga]Ga-FAP-2286 as well as the area of the peak corresponding to free gallium-68 and all other undefined peaks with lower retention times.

For the evaluation of the incorporation of the radionuclide, iTLC was performed using Agilent iTLC-SG, 9 × 1 cm chromatographic paper (Agilent, CA, USA), and a 1:1 mixture of 1 M ammonium acetate and methanol as the mobile phase. The percentage of free gallium-68 ions and gallium-68 colloids migrating with a retardation factor of less than 0.3 was determined using a Scan-RAM radio-TLC scanner with a PS Plastic/PMT detector (LabLogic Systems, Sheffield, UK).

The overall radiochemical purity, combining both chromatographic methods, was calculated from percentage of [^68^Ga]Ga-FAP-2286 (Y) determined from radio-HPLC analysis and free or colloidal gallium-68 species (Z) from iTLC using the following Formula (1):

overall RCP % = (100 − Z) × Y (1)

pH indicator strips (Merck, Darmstadt, Germany) were used to determine the pH of the final injectable solution.

For the measurement of the total radioactivity and determination of the half-life (*n* = 4), an ISOMED 201 dose calibrator (NUVIA Instruments GmbH, Dresden, Germany) was used. The radionuclide identity was confirmed by gamma-ray spectrometry, and germanium-68 breakthrough (GabiStar, Raytest, Straubenhardt, Germany) was quantitatively determined after release following a minimum decay time of at least 48 h.

For the evaluation of the HEPES content, a standard stock solution of HEPES of 500 µg/V in water (V being the maximum recommended volume in mL for administration) according to Ph. Eur. monographs mentioned above was prepared.

After release, endotoxin levels and sterility were tested by accredited laboratories (Mikrobiologisches Prüflabor GmbH, Innsbruck, Austria) according to the European Pharmacopoeia.

For the determination of the total peptide content, a UV-Vis HPLC calibration curve (6 dilutions) was plotted using a GMP vial with defined peptide content (50 µg) dissolved in water (1 mg/mL) (R^2^ = 0.993; limit of quantification < 2.5 µg/mL; signal-to-noise ratio = 14.0 when 0.025 µg was injected.)

The stability of the final product was tested by additional prepared batches in radio-HPLC and iTLC analysis up to 3 h post-production (p.p.) (*n* = 3). The final preparation was stored in a closed lead shielding in an upright position at room temperature.

To further investigate the stability of [^68^Ga]Ga-FAP-2286 during radiolabeling, and possibly to prevent the formation of the hydrophilic impurities, the radiolabeling temperature was lowered to 95 °C. The addition of ethanol (20% *v*/*v* final concentration in the labelling solution) or the addition of ascorbic acid (10 mg or 50 mg) to the reactor and to the final product vial (300 mg) was also investigated by radio-HPLC analysis.

### 4.4. Patient History

A 63-year-old male patient presented initially with post-hepatic cholestasis. An extramurally performed CT scan indicated a tumor centrally in the bile duct system. The patient then developed sepsis with multi-organ failure. Endoscopic retrograde cholangiopancreatography (ERCP) was performed to regain drainage of the bile system for both hepatic lobes. However, several attempts to prove a tumor as the cause of the bile duct stenosis via cytology and biopsy during the ERCPs failed.

A month later, a control CT scan of the abdomen showed no evidence of a bile duct tumor. Yet, a marked increase of CEA and CA19-9 remained highly suggestive of a malignant tumor as the initial cause of the icterus.

An [^18^F]FDG-PET/CT scan was performed and could still not clearly detect any suspicious lesion. Therefore, we suggested an additional [^68^Ga]Ga-FAP-2286-PET/CT scan. In Figure 7, a comparison between the [^18^F]FDG and initial [^68^Ga]Ga-FAP-2286 scans is shown.

### 4.5. [^68^Ga]Ga-FAP-2286 PET/CT Imaging

For PET/CT-imaging, an activity of 2 MBq (range 1.8–2.5 MBq) [^68^Ga]Ga-FAP-2286 per kg body weight was injected intravenously. PET/CT-imaging was performed as a single time-point acquisition 60 min after injection (allowed range from 50 to 70 min). A Biograph 128 Vision 600 Edge PET/CT system (Siemens Healthineers, Erlangen, Germany) was used. The CT scan was performed as a low-dose, non-contrast-enhanced CT scan for attenuation correction and anatomical correlation of PET findings (100 kV, CARE Dose4D dose modulation). For PET acquisition, a continuous bed motion was used with an imaging speed of 0.8 cm/s in craniocaudal direction, resulting in a scan time of 13 min from the base of the skull to the mid thighs. PET image reconstruction was performed as OSEM 3D including TOF information, with four iterations and five subsets, using a Gauss 2.0 filter.

### 4.6. Image Interpretation

A board-certified nuclear-medicine physician evaluated the reconstructed PET, and a board-certified radiologist evaluated the low-dose CT scan. A positive tumor lesion on the PET scan was defined as a focus of activity with an SUV higher than the background activity of the gluteal muscles and not attributable to physiologic distribution such as urinary excretion. Non-focal uptake in organs or structural changes higher than muscle background activity were also noted but not assessed as a malignant tumor. A volume of interest was semi-automatically placed around each uptake of interest, and the calculated SUV_max_ and SUV_peak_ were recorded.

## 5. Conclusions

The peptide-based radiopharmaceutical [^68^Ga]Ga-FAP-2286 was successfully synthesized automatically with minor manual interventions using the commercially available GRP-3V synthesis module, cassette and reagent kit combined with a GalliAd ^68^Ge/^68^Ga generator. All quality parameters tested, including radiochemical purity, pH, endotoxin, sterility and HEPES content comply with the European Pharmacopoeia monographs of other gallium-68 labeled radiopharmaceuticals. Thus, [^68^Ga]Ga-FAP-2286 can be easily and reliably produced for clinical routine. The [^68^Ga]Ga-FAP-2286 images had, in this specific case, a high clinical impact for initiation as well as for the change of systemic chemotherapy in this case, illustrating the power of [^68^Ga]Ga-FAP-2286-imaging in [^18^F]FDG-negative tumors. However, more patients and studies are needed to show a significant benefit for the patients with inconclusive [^18^F]FDG scans.

## Figures and Tables

**Table 1:** Batch analysis of the automated synthesis of [^68^Ga]Ga-FAP-2286. Data are presented as mean ± SD.

Parameter	Method	Acceptance Criteria	Results (*n* = 5)
Appearance	Visual inspection	Clear, colorless solution, free of visible particles	conforms
pH	Indicator strip	4.0–8.0	~7
Volume	Graduated vial	17 mL (range 17 ± 1 mL)	conforms
Radionuclide identity	Gamma-ray spectrometry	511 keV; 1022 keV	conforms
Radionuclide identity	Half-life	1.03–1.23 h	1.13 ± 0.01 h
Identity of [^68^Ga]Ga-FAP-2286 (comparison with reference)	HPLC	RRT 0.9–1.1	conforms
Radiochemical purity	HPLC	≥95%	96.2 ± 1.4%
Free or colloidal gallium-68 (retardation factor < 0.2)	iTLC	≤3%	<1%
Overall radiochemical purity	overall RCP % = (100 − Z) × Y	≥92%	96.1 ± 1.2%
Radiochemical yield	Decay-corrected; calculated	≥70%	71.8 ± 1.9%
FAP-2286 and [^68^Ga]Ga-FAP-2286	HPLC	≤50 µg	42.0 ± 7.6 µg
Ethanol content	Visual inspection, calculated	Volume > 10 mL	conforms
Filter integrity test	Bubble-Point test	>3.5 bar	conforms
Radionuclide purity	Gamma-ray spectrometry	Ge-68: ≤0.001% (after ≥48 h)	conforms
Bacterial endotoxins	LAL test	≤11 EU/mL	<11 EU/mL
Sterility	Ph. Eur.	sterile	conforms
HEPES content	Visual; Ph. Eur.	<500 µg/V	conforms

**Table 2:** Stability data of [^68^Ga]Ga-FAP-2286 up to 3 h after preparation. Data are presented as mean ± SD.

	Acceptance Criteria	p.p.	1 h	2 h	3 h
% RCP (HPLC)	≥95%	97.8 ± 0.7%	98.0 ± 0.3%	98.6 ± 0.5%	98.8 ± 0.1%
% colloidal gallium-68 species	≤3%	≤1%	≤1%	≤1%	≤1%

**Table 3:** Results from changes during the radiolabeling process.

	Acceptance Criteria	95 °C	20% Ethanol	10 mg AA	50 mg AA
% RCP (HPLC)	≥95%	98.4%	95.3%	97.9%	98.6%
% colloidal gallium-68 species	≤3%	≤1%	≤1%	≤1%	≤1%
pH	4.0–8.0	~7	~7	~7	~7

## Data Availability

The data is contained in the main text and Supplementary Materials.

## Supplementary Materials

The following supporting information can be downloaded at: https://www.mdpi.com/article/10.3390/ph17091141/s1; Figure S1: UV-Vis HPLC chromatogramm of FAP-2286; Figure S2: UV-Vis HPLC chromatogramm of [^non-radioactive^Ga]Ga-FAP-2286; Figure S3: Electrospray ionization mass spectrometry of [^non-radioactive^Ga]Ga-FAP-2286; Figure S4: Radio-HPLC chromatogram of [^68^Ga]Ga-FAP-2286 (a) post production and (b) post production with addition of H_2_O_2_; Table S1. Results from the single batch analysis; Figure S5. Magnified radio-HPLC chromatograms of [^68^Ga]Ga-FAP-2286, showing the hydrophilic impurities with different modifications during radiolabeling: (a) 10 mg ascorbic acid + 300 mg ascorbic acid, (b) 50 mg ascorbic acid + 300 mg ascorbic acid, (c) addition of ethanol (20% *v*/*v* final concentration) and (d) reducing the labeling temperature to 95 °C; Figure S6. Magnified UV-HPLC chromatograms of [^68^Ga]Ga-FAP-2286.
