# Supplementary material for: [68Ga]Ga-FAP-2286—Synthesis, Quality Control and Comparison with [18F]FDG PET/CT in a Patient with Suspected Cholangiocellular Carcinoma
Source: Pharmaceuticals (Basel). 2024 Aug 29;17(9):1141. doi: 10.3390/ph17091141 (PMC11435094; doi:10.3390/ph17091141)
Supplement: Supplementary file 1 [file pharmaceuticals-17-01141-s001.zip › pharmaceuticals-3158402-supplementary.pdf]

[^{68}Ga]Ga-FAP-2286 – Synthesis, Quality Control and Comparison with [^{18}F]FDG PET/CT in a Patient with Suspected Cholangiocellular Carcinoma

Anton Amadeus Hörmann [1], Gregor Schweighofer-Zwink [1], Gundula Rendl [1], Kristina Türk [1], Samuel Nadeje [1], Theresa Jung [1], Ursula Huber-Schönauer [1], Lukas Hehenwarter [1], Mohsen Beheshti [1] and Christian Pirich [1]

1 Department of Nuclear Medicine and Endocrinology, University Hospital Salzburg, Paracelsus Medical University, Salzburg, Austria

Table of Contents

Figure S1: UV-Vis HPLC chromatogram of FAP-2286
Figure S2: UV-Vis HPLC chromatogram of [$^{\text{non-radioactive}}$Ga]Ga-FAP-2286
Figure S3: Electrospray ionization mass spectrometry of [$^{\text{non-radioactive}}$Ga]Ga-FAP-2286
Figure S4: Radio-HPLC chromatogram of [^{68}Ga]Ga-FAP-2286 (a) post production and (b) post production with addition of H_2O_2
Table S1. Results from the single batch analysis
Figure S5. Magnified radio-HPLC chromatograms of [68Ga]Ga-FAP-2286, showing hydrophilic impurities with different modifications during radiolabeling: (a) 10 mg ascorbic acid + 300 mg ascorbic acid, (b) 50 mg ascorbic acid + 300 mg ascorbic acid, (c) addition of ethanol (20% v/v final concentration) and (d) reducing the labeling temperature to 95 °C
Figure S6. Magnified UV-HPLC chromatograms of [^{68}Ga]Ga-FAP-2286

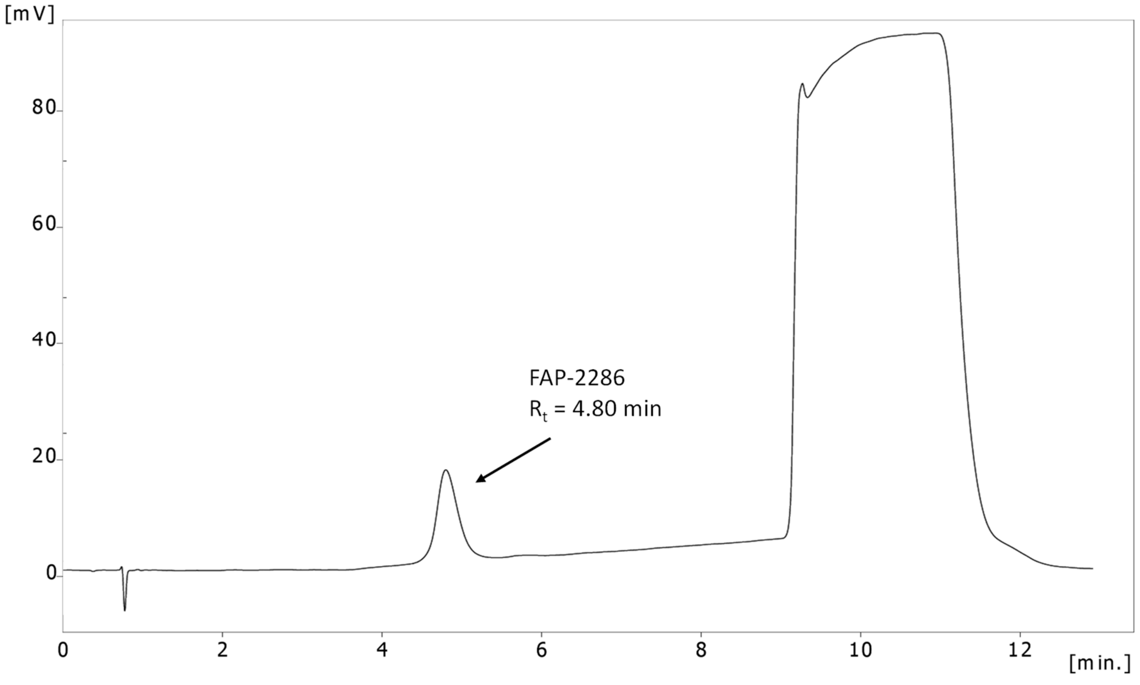

Figure S1. UV-Vis HPLC chromatogramm of FAP-2286

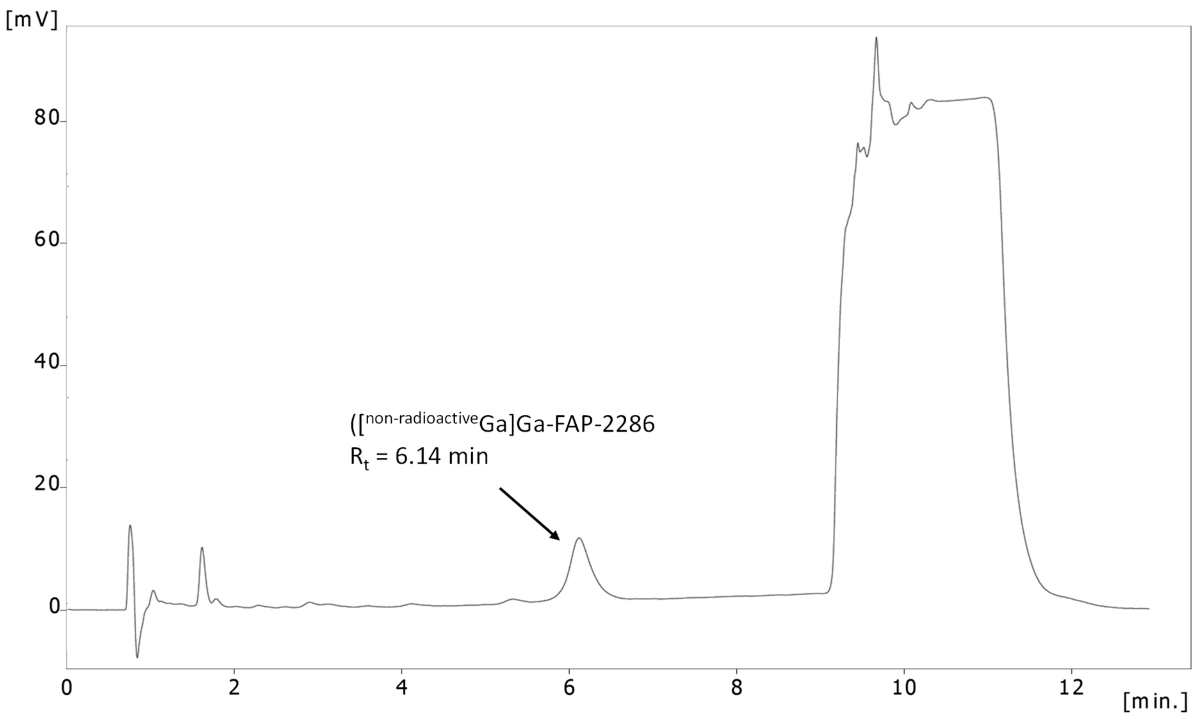

Figure S2. UV-Vis HPLC chromatogramm of ([non-radioactive Ga]Ga-FAP-2286

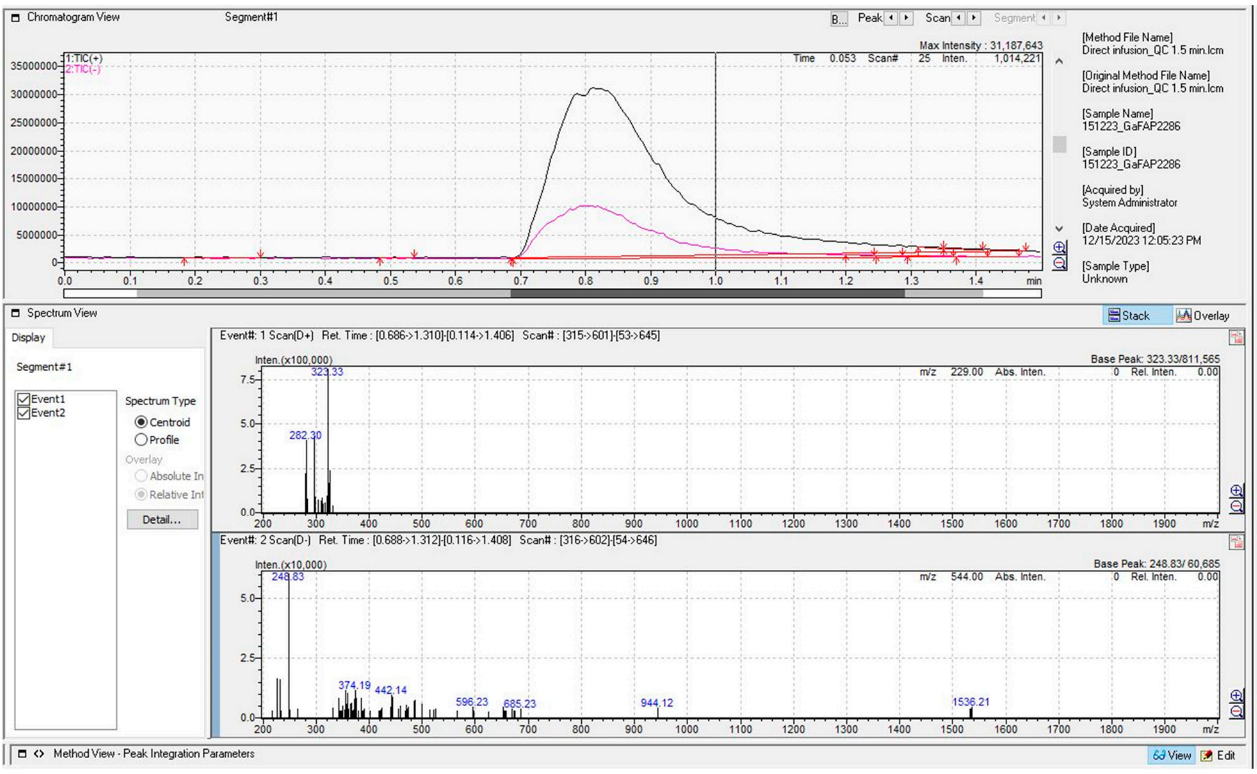

Figure S3. Electrospray ionization mass spectrometry of [non-radioactive Ga]Ga-FAP-2286

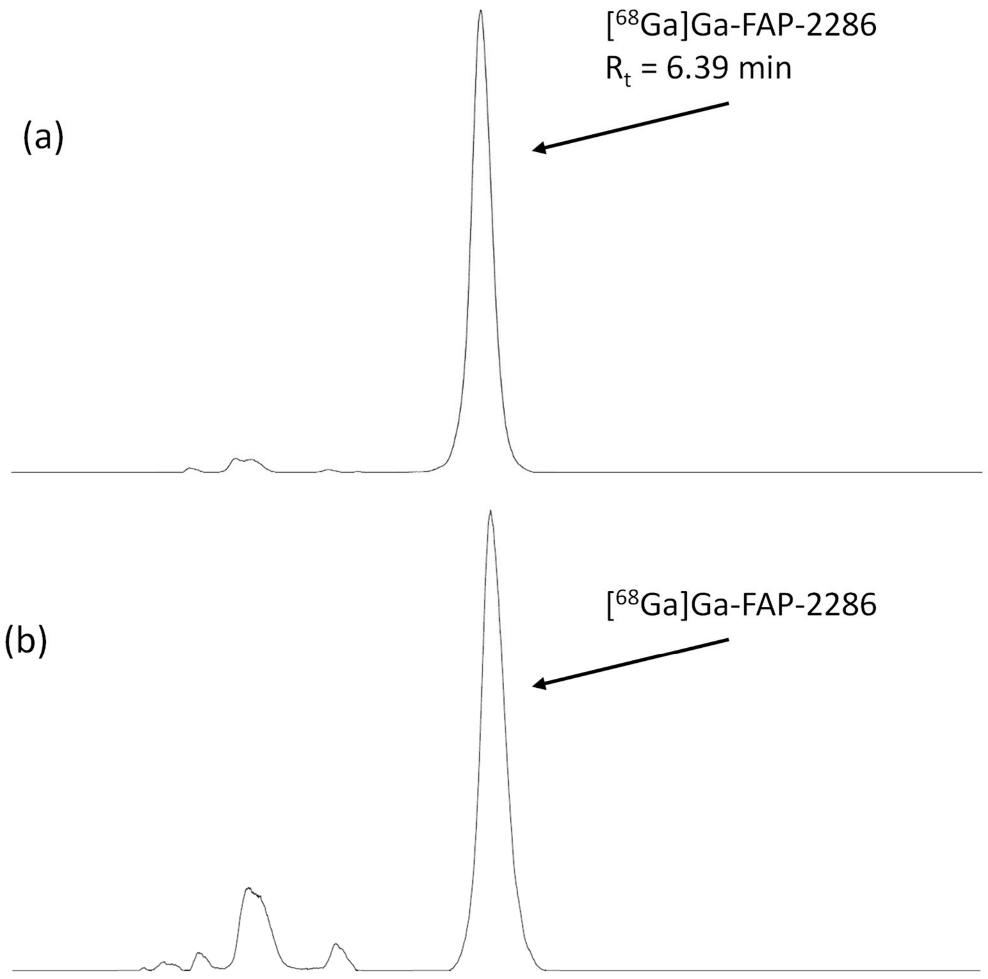

Figure S4. Radio-HPLC chromatogram of [^{68}Ga]Ga-FAP-2286 (a) post production and (b) post production with addition of H_2O_2

Table S1. Results from the single batch analysis

Batch number	**Acceptance criteria**	1	2	3	4	5
Overall radiochemical purity	≥92%	95.7%	96.3%	94.5%	98.2%	95.7%
Radiochemical yield d.c.	≥70%	69.7%	73.0%	70.1%	74.7%	71.6%

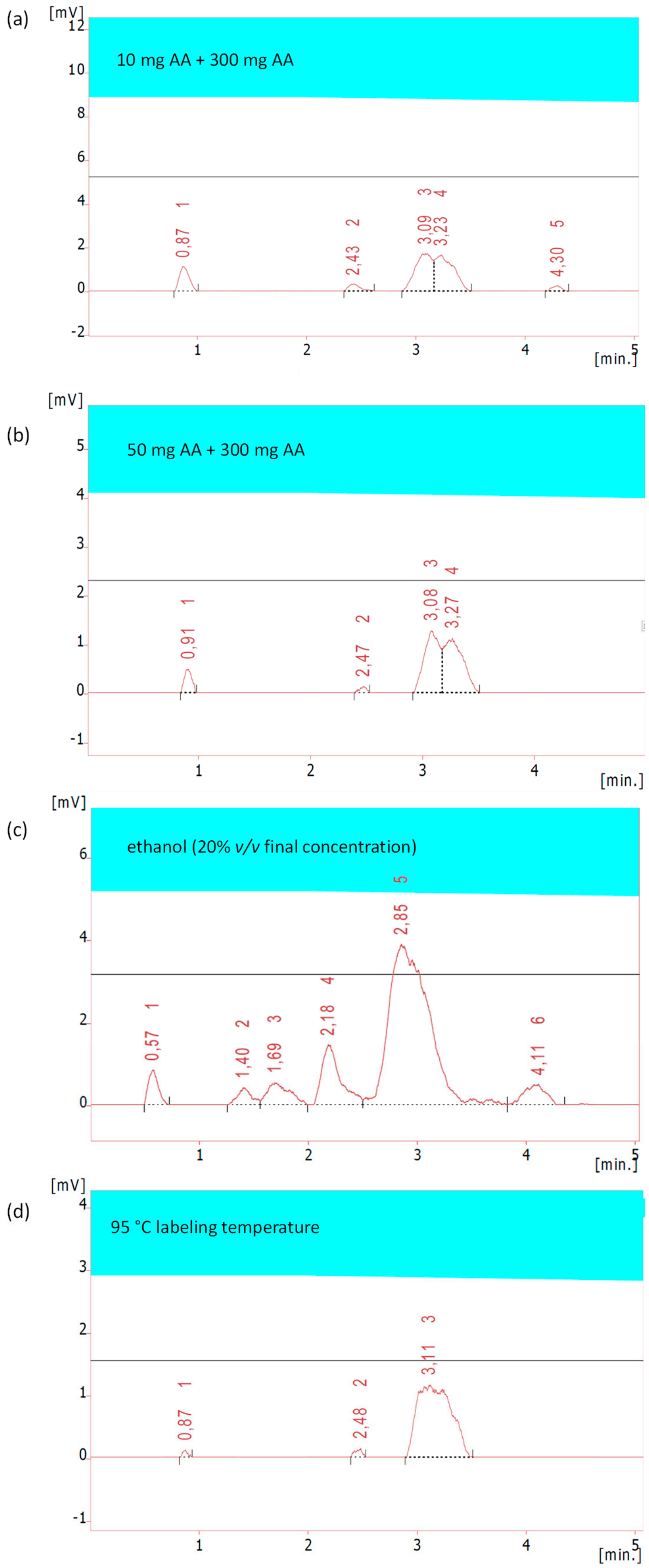

Figure S5. Magnified radio-HPLC chromatograms of [^{68}Ga]Ga-FAP-2286, showing hydrophilic impurities with different modifications during radiolabeling: (a) 10 mg ascorbic acid + 300 mg ascorbic acid, (b) 50 mg ascorbic acid + 300 mg ascorbic acid, (c) addition of ethanol (20% v/v final concentration) and (d) reducing the labeling temperature to 95 °C

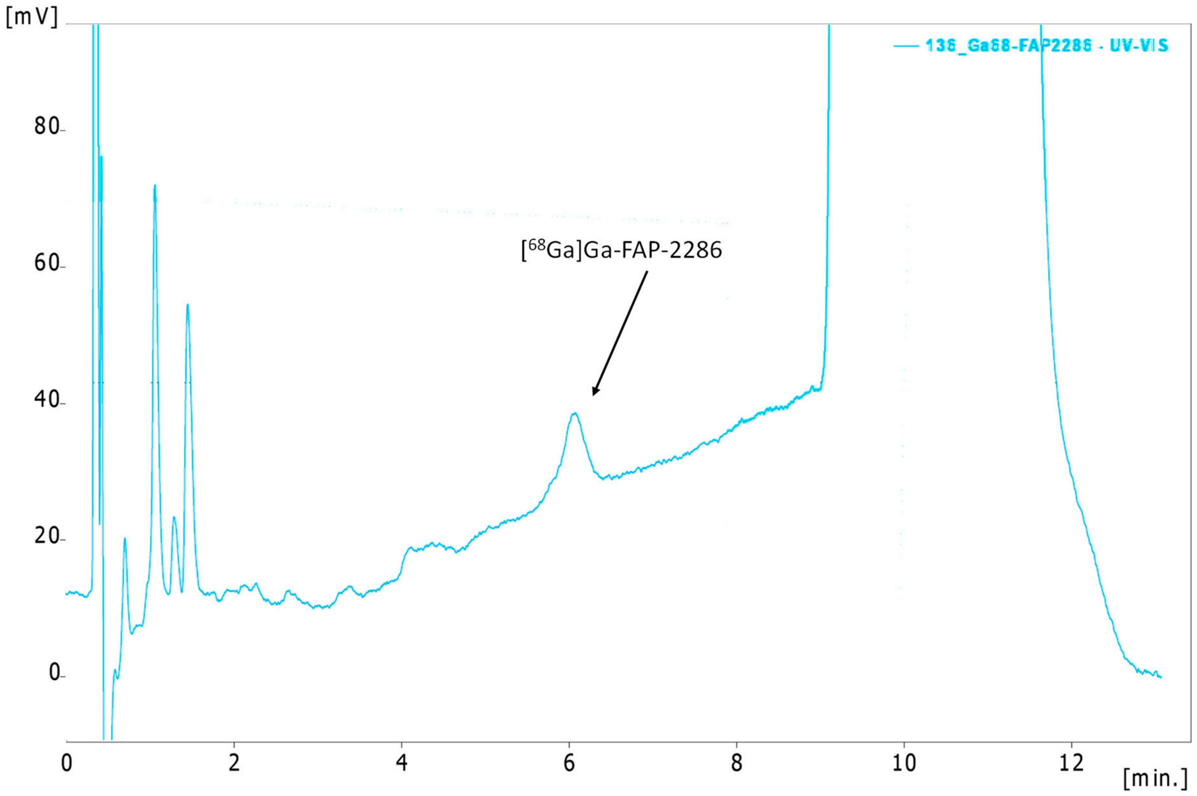

Figure S6. Magnified UV-HPLC chromatograms of [^{68}Ga]Ga-FAP-2286
